# Impacts of Environmental Regulations on Tourism Carbon Emissions

**DOI:** 10.3390/ijerph182312850

**Published:** 2021-12-06

**Authors:** Qiao Chen, Yan Mao, Alastair M. Morrison

**Affiliations:** 1 School of Tourism and Hospitality Management, Hubei University of Economics, Wuhan 430205, China; chenqiao@hbue.edu.cn; 2 Department of Marketing, Events and Tourism, Greenwich Business School, University of Greenwich, London SE10 9SL, UK; a.morrison@greenwich.ac.uk

**Keywords:** environmental regulations, tourism carbon emissions, public participation, command and control, market incentives, supervisory management

## Abstract

This research analyzed the impact of environmental regulations and their power in suppressing tourism carbon emissions. The results showed that: (1) four types of environmental regulations had significant inhibiting effects on tourism carbon emissions, but different types of regulations had varying effects; and (2) environmental regulations had a significant time lag effect on tourism carbon emissions. The decay rates of the environmental regulation effects were dissimilar for supervisory management, market incentives, command and control, and public participation; and (3) environmental regulations had dissimilar influences on tourism carbon emissions at the regional level. Government agencies should choose differentiated environmental regulation tools, attach great importance to the time-lag effect of environmental regulations on tourism carbon emissions, and establish systems and mechanisms of public participation in environmental matters.

## 1. Introduction

Over the past 40 years of reform and opening-up, China’s tourism economy has maintained rapid growth with an annual average rate of over 15%. However, in contrast to the common perception that tourism is a smokeless green industry, it has become a significant carbon emitter. In 2005, China’s tourism carbon emissions were 132.74 million tons, accounting for 2.41% of China’s total carbon emissions. In 2020, China’s tourism carbon emissions were 4738.64 million tons, accounting for 4.93% of China’s total carbon emissions. During 2005–2020, the tourism industry’s carbon emissions of China increased at an average annual rate of 2.31% [1,2]. Furthermore, according to the 2018 Global Environmental Performance Index (GEI) released by Yale University and other authoritative institutions, China’s air quality ranked fourth from the bottom, trailing only India, Bangladesh and Nepal. In China, tourism carbon emission reduction is imperative considering the rapid tourism growth. The high carbon emissions and serious environmental pollution of the tourism sector present a dilemma in achieving stable and long-term development. China has actively formulated relevant policies and adopted environmental regulations to control tourism carbon emissions to upgrade the industry structure and promote the low-carbon transformation of tourism. However, several research questions remain unanswered. These are: can environmental regulations become important driving forces in promoting low-carbon tourism? What are the differences in the effects of different types of environmental regulations on tourism carbon emissions? Whether environmental regulations have different effects on carbon emission reduction of tourism in different regions? Are there lag effects of environmental regulations on carbon emissions?

## 2. Literature Review

Several scholars have studied the impacts of environmental regulations on carbon emissions. However, differing views on the effects of environmental regulations on carbon emissions have emerged, and these can be divided into three categories. The first notion is that environmental regulations promote carbon emissions, which Sinn (2008) termed the concept of the “green paradox.” With increasingly strict environmental regulations, he believed that energy developers accelerate the exploitation of fossil energy, leading to an increase in carbon emissions, and this is not conducive to improving carbon emission efficiency [3]. Governments often enact macro policies to curb carbon emissions and these policies impact carbon emission intensity. Environmental regulations improve carbon emission efficiency, and the effect is pronounced [4]. Additionally, environmental regulations effectively improve per capita carbon emission efficiency, but there are regional differences [5]. In addition, environmental regulations have a spatial correlation effect on carbon emissions [6,7,8]. Zhang (2014) studied the impacts of environmental regulations on carbon emissions based on different spatial weight matrices and found that environmental regulations in the subject and neighboring regions significantly promoted carbon emissions [9]. Environmental regulations reduced carbon emission efficiency, and the “green paradox” was established [10].

The second school of thought is that environmental regulations curb carbon emissions. Some scholars believe that environmental regulations have a “forced effect” on carbon emissions. Agriculture is an important industry of the national economy, through measures such as banning straw burning, environmental regulations have had a significant moderating effect on the impact of agricultural carbon emissions. Still, this effect existed only in the eastern region [11]. However, different types of environmental regulations had dissimilar impacts on resource allocations in the market, and thus had varying inhibitory effects on carbon emission efficiency [12]. Environmental regulations can divide into cost and investment types, and both have restraining effects on tourism carbon emissions [13]. For different industries, the effect of environmental regulations on carbon emission suppression may also be different. Some scholars found a negative correlation existed between environmental regulations and tourism carbon emissions, and environmental regulations had more of an inhibitory effect on transportation carbon emission than for accommodation [13,14,15].

The third proposition is that there is no linear relationship between environmental regulations and carbon emissions, and an increase in environmental regulation intensity does not reduce the carbon emissions of tourism [16]. These scholars propose that environmental regulations and carbon emissions do not inhibit or promote the relationship and there may be an inflection point phenomenon [12]. For different income levels, the impact of environmental regulations on carbon emissions may have nonlinear characteristics [17,18]; environmental regulations do not promote carbon emissions in the middle and low threshold ranges, while energy intensity promoted carbon emissions in the high threshold range [19]. Yang (2020) used the intensity of environmental regulations as a threshold variable to build a threshold regression model and found that different intensities of environmental regulations had a promoting effect on carbon emissions. However, this effect increased with an increase in the intensity of environmental regulations [20]. There may be single or double thresholds for environmental regulations to carbon emissions, and the effects of environmental regulations on carbon emissions are different under dissimilar thresholds [21,22]. Yang and Wang (2021) determined that cost-type environmental regulations had a single threshold value for tourism carbon emissions, while investment-type environmental regulations had a double threshold effect [23]. Some scholars believe that the relationship between environmental regulations and carbon emission efficiency is “U-shaped” [24]. That is, with an increase of environmental regulation intensity, the leading force of its influence changes from the “green paradox” to “forcing emission reduction” [25].

In summary, environmental regulations have been analyzed from multiple perspectives in terms of their influence on carbon emissions. However, there are still some gaps in tourism-related research: several scholars have conducted studies on environmental regulation and industrial carbon emissions, but there are few studies on impacts on tourism carbon emissions. However, the contribution of tourism to carbon emissions must not be ignored. It is imperative to measure the effect intensity of environmental regulations on tourism carbon emissions. Such measurement research will contribute to the realization of global tourism carbon emission reduction as expressed in the United Nations Sustainable Development Goal 13 (Climate Action). The current description of environmental regulation indicators is not comprehensive enough. The selection of environmental regulation indicators remains relatively simple and the resulting research conclusion may be too one-sided [26]. There is a lack of research on the differences in the effects of various environmental regulation tools on tourism carbon emissions. Therefore, this research divided environmental regulation into four types: command and control, supervisory management, market incentives, and public participation. Based on panel data of the regulation intensity index from 1999–2019, the differing effects of various environmental regulations on tourism carbon emissions were measured. The following questions were addressed: can various environmental regulations restrain tourism carbon emissions? How strong are the restraining actions and for how long do they persist? Are there differences in the intensity of actions by regions?

This study not only theoretically clarifies the impact of environmental regulations on tourism carbon emissions; it will be helpful in improving the implementation of environmental regulations. It is of practical value in promoting environmental improvements and the high-quality development of the tourism economy. The potential contributions of the research findings are as follows. First, a new four-dimensional environmental regulation index system was developed to evaluate the inhibitory effects of environmental regulations on tourism carbon emissions. Second, the lag effects of four types of environmental regulations on tourism carbon emissions were examined, as was the duration of the impacts of environmental regulations. Third, a spatial dimension analysis demonstrated the differing effects of the four types of environmental regulations on regions.

## 3. Analysis of Impacts of Environmental Regulations

Scholars have done substantial research on the impacts of environmental regulations on selected industries. However, there is scant empirical evidence on the influence of environmental regulations on tourism carbon emissions. The effects of environmental regulations on tourism carbon emissions can be categorized into four types.

First, command control environmental regulation restricts tourism carbon emissions by governmental agencies legislating and formulating rules and regulations, requiring enterprises to comply with them by administrative orders, and punishing those that violate relevant standards. Command and control environmental regulation forces companies to comply with the relevant environmental regulations [27,28]. Source control is through the formulation of relevant laws and regulations to improve environmental standards and restrict the entry of polluting enterprises [29]. Terminal management is to affect the production costs of enterprises by means of administrative punishments [30], order rectification, or closure according to the relevant laws and regulations.

Market incentive environmental regulation to reduce emissions encourages or penalizes actions through subsidies or penalties. This is accomplished through fiscal tax measures, financial, and other economic measures [31,32]. Subsidies, grants, discounted interest rates and preferential credit, and other rewards are given to companies for energy conservation, pollution reduction and other beneficial actions [33]. Tax and other penalties are levied on violators.

The restraining effects of supervisory management environmental regulations are through organizing and formulating environmental protection plans, coordinating environmental protection work for tourism and its policies and legislation, and inspecting, supervising and guiding the implementation of environmental protection laws [34] by relevant government departments and tourism companies.

The effects of public participation environmental regulation is through publicity and changing public opinion that promotes participation in the formulation of environmental regulations and guides individuals or companies to save energy and reduce emissions. People are increasingly expressing environmental demands to relevant administrative organizations to safeguard their rights and interests when environmental pollution damages their interests [35]. Citizens provide feedback on the carbon emissions of tourism enterprises to government agencies. They use Internet platforms, complaint letters, petitions, and other means to report environmental demands to the relevant government departments [36], and then these agencies impose administrative control on pollution-intensive enterprises [37]. Polluters who cause property losses are ordered to pay compensation in accordance with the law, and polluting behavior is subject to administrative punishment. Additionally, people share carbon emission information on tourism enterprises through public opinion media [38]. This exposure negatively affects the market images of pollution-intensive enterprises, resulting in a reduction of intangible assets, forcing companies to reduce emissions and pollution, or move to areas with weaker environmental regulations.

## 4. Model Construction and Data

### 4.1. Model Setting

Referring to Chen et al. (2019) [39] and Yang et al. (2021) [23], environmental regulations may have an “individual effect” on tourism carbon emissions that does not change over time. There may also be “time effects” that do not vary with individual differences [40]. A two-way fixed-effect model was designed to test the correlations between environmental regulations and tourism carbon emissions to fully reveal the impacts. The model structure is shown in Equation (1).

(1) $$TCE_{it}=\beta_{0}+\beta_{1}ER_{it}+\sum \beta X_{Control}+\sigma_{t}+\mu_{i}+\varepsilon_{it}.$$

where i epresents each province, t represents the year, $TCE_{it}$ stands for tourism carbon emissions (response variable) and $ER_{it}$ is the environmental regulations (explanatory variable). The environmental regulations were divided into four categories: command and control, supervisory management, market incentives, and public participation. $X_{Control}$ represents a series of control variables, $\sigma_{t}$ represents a time effect that does not vary with individual differences, $\mu_{i}$ is the individual effect that does not change with time, and $\varepsilon_{it}$ is the random error term.

### 4.2. Variable Descriptions

#### 4.2.1. Response Variable

Tourism carbon emission ($TCE_{it}$): the annual tourism carbon emissions of each province were selected as the index to assess the scale of carbon emissions [41]. Based on the System of National Accounting and the energy balance table in the China Energy Statistical Yearbook, the calculation used the “top-down” method, used by Xie et al. (2012) [42] and Huang et al. (2021) [43]. The specific formula was as follows:

$$D_{i}=T_{i}X_{i}$$

$$E_{it}=E_{ij}D_{i}$$

$$TCE_{it}=\overset{n}{\underset{i=1}{\sum }}\left(E_{ij}f_{j}k\right)$$

where $D_{i}$ is the stripping coefficient of tourism consumption, $T_{i}$ is the added value of the three industries, and $X_{i}$ represents the added value of tourism in the three industries. $E_{ij}$ represents the amount of *j* energy consumed by industry *i*; $E_{it}$ represents the total amount of tourism-related energy consumption in three industries, $f_{j}$ represents the standard coal conversion coefficient of j energy, k represents carbon dioxide emissions per unit of standard coal, and k is set to 2.45 [39].

#### 4.2.2. Explanatory Variables

Referring to Wei and Liu (2014) [44], Wang et al. (2018) [19], and Yang et al. (2021) [23], environmental regulations were separated into four types: command and control, supervisory management, market incentives, and public participation.

(1) Command control environmental regulations (CC): the government issues or promulgates laws and regulations, and normative documents, etc., requiring enterprises to strengthen environmental protection and reduce carbon emissions [45,46,47]. For example, with “environmental protection” as the keyword, 31,725 regulatory documents issued by various provinces and cities from 1999–2019 were retrieved from the PKULAW database. There were 621 local laws and regulations, 178 local government regulations, 7463 local normative documents, and 23,463 local working documents.

(2) Market incentives environmental regulations (MI): governments strengthen the guidance of environmental pollution control through subsidies or penalties and increases the investment in environmental pollution control to reduce pollution discharge [48]. In this research, the investment amount of environmental governance in each province was derived from the economic and investment environmental regulation index.

(3) Supervisory management environmental regulations (SM): refers to programs by the government to reduce pollution by supervising pollutants discharged by enterprises [49,50]. It is an ex post surveillance mechanism. In this research, the number of environmental monitoring staff in each province was used as a measurement index.

(4) Public participation environmental regulations (PP): this is the promotion of public participation in carbon emission supervision and governance through publicity and public opinion gathering and guiding individuals and enterprises to save energy and reduce emissions [51]. Scholars use numbers of environmental letters and visits, volumes of environmental visitors, numbers of environmental pollution news reports, complaints, and other indicators for measurement [52,53]. However, some scholars used a comprehensive measurement index [54]. The number of environmental petitioners was used to measure public participation in environmental regulations in this research.

#### 4.2.3. Control Variables

Tourism income (TI) was the total income earned by enterprises providing facilities, goods, and services to tourists [55]. The higher the tourism income, the greater the carbon emission. Employment in the tertiary industry (ET) was the jobs in services such as wholesaling and retailing, accommodation and catering, culture, sports, and entertainment [56]; transport passenger volume (PV) is an index measuring the provincial transport infrastructure network volumes and tourism accessibility [57]. The more accessible the transportation, the more readily it attracts tourists.

### 4.3. Data Sources

This study considered 31 provinces of China excluding Hong Kong, Macao, and Taiwan, for which there was a lack of data. The tourism carbon emission ($TCE_{it}$), tourism income (TI) and revenue data were obtained from the China Energy Statistical Yearbook from 2000–2020. Data on provincial environmental pollution control investment, employment in the tertiary industry (ET) and transport passenger volume (PV) were obtained from the EPS (China Microeconomic Data Query System) database (http://olap.epsnet.com.cn, 8 June 2021). Command control environmental regulations come from PKULAW (Peking University Fabao Database) database (https://www.pkulaw.com/law/adv/lar, 8 June 2021). Market incentives environmental regulations came from the EPS database. Data on supervisory management environmental regulations were obtained from Provincial Statistical yearbooks. Data on public participation environmental regulations were obtained from the China Environmental Statistics Yearbook (Table 1).

## 5. Empirical Tests

Based on the panel data of 31 provinces in China from 1999–2018, OLS, FE, and RE were constructed from the panel data, and the F-and Hausman tests were used to investigate the models. A fixed-effect model was selected to empirically test the impacts of environmental regulations on tourism carbon emissions.

### 5.1. Impacts of Environmental Regulations on Tourism Carbon Emissions

Table 2 shows the impacts of the four environmental regulations on tourism carbon emissions. The impact of command and control environmental regulations (CC) on tourism carbon emissions was 0.16 at the 1% significance level, indicating that governments can effectively reduce the carbon emission of tourism enterprises by issuing administrative instructions or formulating laws and regulations for tourism operations. The influence coefficient of market incentives environmental regulation (MI) on tourism carbon emission was −2.64, significant at the 1% level. The effect on carbon emissions based on supervisory management environmental regulations (SM) was 1.60, significant at the 1% level. The influence coefficient for the public participation environmental regulation (PP) effect on tourism carbon emission was −0.07, which was significant at the 1% level (Table 2).

There were differences in the effects of various environmental regulations on tourism carbon emissions. Among the four types, market incentives environmental regulation had the largest impact coefficient on tourism carbon emissions (−2.64), while public participation environmental regulation had the lowest impact coefficient (−0.07). Environmental regulations based on market incentives are the most direct carbon emission reduction measure. They increase investment in carbon emission controls and purchases of more advanced emission control equipment, and improve tourism pollution control capacity to meet government carbon emission reduction standards. Market incentive environmental regulations (MI) is the most direct and effective measure to reduce the carbon emissions of tourism enterprises. The government encourages tourism companies to reduce carbon emissions through preferential policies and increased investment. In contrast, public participation environmental regulations (PP) rely on people’s environmental awareness and attention to the environment and urges enterprises to reduce carbon emissions through public supervision, which is not legally enforceable and binding. Therefore, the influence coefficient of public participation in environmental regulation on tourism carbon emission is much lower than that of market incentives. The influence coefficient for supervisory management environmental regulation on tourism carbon emissions was second only to that of market incentives. Supervisory management environmental regulations can have an “immediate effect” on tourism carbon emissions, and strong policy guidelines rapidly affect corporate tourism activities. However, supervisory management environmental regulations are a post-treatment measure, often treating the symptoms rather than the root causes of tourism carbon emissions. If there is a lack of awareness of supervision, the impact on tourism carbon emissions can be minimal. Among the four types of regulations, public participation had the least influence on tourism carbon emissions. Most urban citizens have limited awareness of tourism carbon emission reduction and emission supervision [58]. Public participation in tourism carbon emissions still has scope for enhancement and improvement. Other control variables, including the number of employees in tertiary industries, tourism income, and transportation accessibility, had significant inhibitory effects on tourism carbon emissions.

### 5.2. Robustness Test

The robustness of the model was tested by comparing various balanced short panel data models. According to the established index data, the mixed regression, fixed effect, random effect models, and inter-group estimators were established, denoted as OLS, $FE_{robust}$, $FE_{TW}$, RE, and BE. As the clustering robust standard error is larger than the ordinary standard error and more accurate for model testing results, this research adopted the clustering robust standard error. The premise of the mixed panel data regression model was that all individuals behave in the same way at all times [59]. The fixed-effect model is a statistical model that represents the observed quantities in terms of explanatory variables that are treated as if the quantities were non-random. It controls for individual-level time-invariant factors. The random-effect model considered the “individual effect” as a random factor. The intergroup estimator was a time average for each individual, and this average was used for regression analysis. The regression results for each model are shown in Table 3, and the coefficient estimates of different regression models were the same as the results of the benchmark regression model. Therefore, it can be concluded that the estimation results are robust.

## 6. Discussion: Time-Lag and Regional Variation

### 6.1. Time-Lag Effects of Environmental Regulations

**Figure 1:**
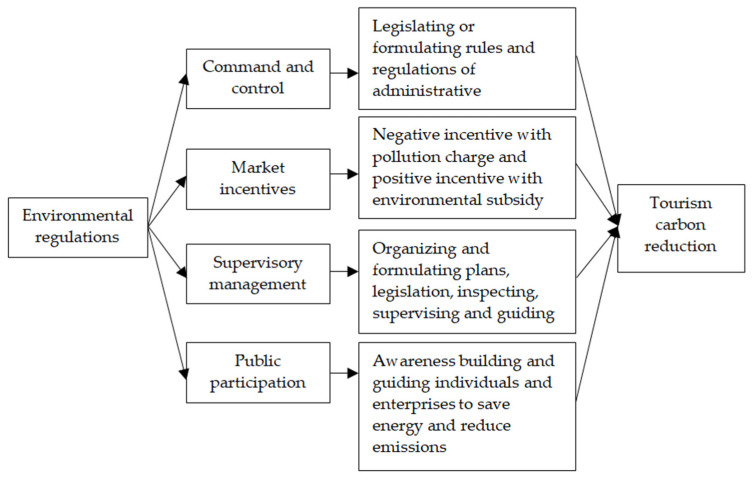
Types of environmental regulations affecting tourism carbon emissions.

Environmental governance and restoration are continuous and lengthy processes. Therefore, the influence of environmental regulation policies on environmental governance should be considered. Environmental regulation measures implemented by provinces may have a strong governance effect in the current year, or they may not have an impact until several years later. Therefore, due to the potential of a lag effect, it may not be possible to accurately and comprehensively evaluate the impacts of environmental regulations on tourism carbon emissions if only the intensity of current environmental regulations is considered. Therefore, the intensity of the environmental regulation with one to five lag periods was substituted into the model (2) as independent variables. The time lag effects of environmental regulations on tourism carbon emissions were further analyzed. The regression results are shown in Figure 1 and Table 4.

$$TCE_{it}=\beta_{0}+\beta_{1}CC_{it-k}+\beta_{1}EI_{it-k}+\beta_{1}SM_{it-k}+\beta_{1}PP_{it-k}+\sum \beta X_{Control}+\sigma_{t}+\mu_{i}+\varepsilon_{it},$$

k=1,2,3,4,5

Table 4 shows that: (1) Due to the low efficiency of policy transmission, the carbon emission reduction laws and regulations promulgated by government take a long time to produce a suppressive effect on tourism carbon emissions. There is a multi-stage impact of command and control environmental regulations on tourism carbon emission reduction. Additionally, command and control environmental regulations are time-sensitive and may be terminated or abolished after some time. Therefore, command and control environmental regulations have a significant lag effect on tourism carbon emissions. The influences of command and control environmental regulations in the first, second, third, and fourth stages were −0.07 at the 1%, −0.06 at the 1%, −0.04 at the 1% and −0.02 at the 5% significance levels, respectively. However, when the command and control environmental regulations were delayed in the fifth stage, they did not significantly inhibit tourism carbon emissions.

(2) For market incentives environmental regulation, different lag periods had varying effects on tourism carbon emissions. The impacts of market incentives lag periods on tourism carbon emissions were in the shape of “~” with a lag of −1.41 in the first stage, a small attenuation in the second, third, and fourth stages (−1.20, −1.19, −1.17), and a significant decline in the fifth stage (−0.9). These economic and investment environmental regulations had significant effects on carbon emission reduction of tourism within four lag periods, but the decline was larger in the fifth lag period. Market incentives appear not to be a long-term solution, but a remedy for environmental governance in the short term. These incentives are not effective if applied just once; they require governments and companies to continuously strengthen investment in carbon reduction.

(3) The impact of supervisory management environmental regulations on tourism carbon emissions presented an “S” shape. When the third period was lagged, these regulations did not significantly impact tourism carbon emissions. The impact was mainly concentrated in the first two periods and was significant at the 5% level. Notably, the supervisory management type had long-term characteristics. Its mode is mainly incentive-based, combined with the coordination of personnel and administrative regulations, producing a long-term inhibition effect on tourism carbon emissions. Especially when policies and regulations are first promulgated, all government departments and enterprises will strictly abide by them. However, as time goes by and new policies are issued, the effectiveness of older policies and regulations will gradually weaken.

(4) For the public participation environmental regulations, the impacts for the first, second and third lag periods on tourism carbon emissions were −0.04, −0.04, −0.03 at the 5%, 1%, and 10% significance levels, respectively. However, in the fourth phase and beyond, they did not significantly inhibit tourism carbon emissions. The effect intensity on tourism carbon emissions was far lower than the other three types of environmental regulations. The possible reason is that public participation in environmental regulation guides behavior in general behavior and is not compulsory [60]. The public participates in the supervision of tourism carbon emissions through telephone and mailed complaints, petitions, and other channels. Government departments visit and investigate the issues after receiving complaints [61]. It is challenging for them to investigate and collect evidence, and the case processing cycle is long. Therefore, the effect on the carbon emissions of tourism is slow and its effect intensity coefficient is small.

**Figure 2:**
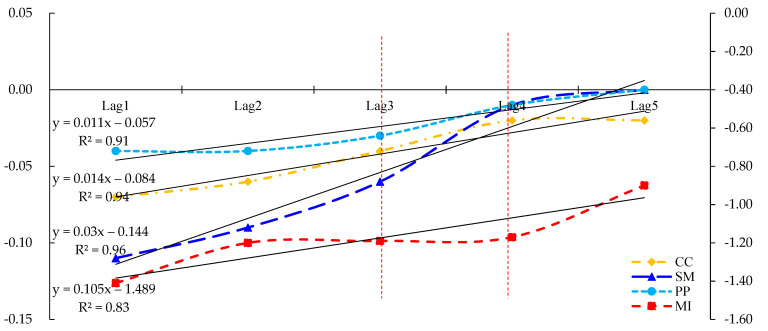
Lag effect of environmental regulation on tourism carbon emissions.

There were differences in the decay rates of various environmental regulations on tourism carbon emissions. As shown in Figure 2, the decay rates of the impacts of the four types of environmental regulations on tourism carbon emissions were market incentives (0.105), supervisory management (0.030), command and control (0.014), and public participation (0.011). Market incentives had an “immediate effect” on tourism carbon emissions. They were also the fastest declining environmental regulation, showing a downward trend in the fifth period. However, the effect intensity of the other three environmental regulation types on tourism carbon emissions, namely supervisory management, command and control, and public participation, showed significant declines in the fourth period. Notably, market incentives had a strong effect on tourism carbon emissions for a longer duration. However, command and control, supervisory management, and public participation all had the characteristics of small effect intensity and short duration. Government departments should select environmental regulations based on the time-effectiveness attributes of various environmental regulations.

### 6.2. Regional Effects of Environmental Regulation

Based on a nationwide research sample, the foregoing results indicate that different types of environmental regulations significantly differ in impacts on tourism carbon emissions. However, are there spatial differences in the effects of environmental regulations on tourism carbon emissions for the eastern, central, and western regions of China? This research investigated the variations in the impacts of environmental regulations on tourism carbon emissions by region (Table 5).

As shown in Table 5, the carbon emissions from tourism in eastern China were affected by the four environmental regulation types. The regression coefficients of command control, market incentives, supervisory management, and public participation on tourism carbon emissions in eastern China were −0.06, −1.69, −0.09 and −0.03, respectively. They were significant at the 1%, 1%, 10% and 10% levels, respectively. This indicates that these four environmental regulations restrict tourism carbon emissions in eastern China. Notably, the influence coefficient of environmental regulation of market incentives type in eastern China is smaller than that in central and western China. Due to the poor economic foundation and weak ability to attract foreign investment in western China, the government has less funds for tourism carbon emission reduction [62,63]. These characteristics make the marginal effect of market incentives environment regulation on tourism carbon emission reduction in western China larger than that in eastern China.

The carbon emissions of tourism in central China were affected by three environmental regulations: command and control, supervisory management, and market incentives. Unlike in eastern China, the impact of public participation environmental regulations on tourism carbon emissions was not significant. In addition, public participation environmental regulation had no significant effect on tourism carbon emission reduction in western China. This may be due to the weak awareness of carbon emission reduction, environmental protection [64], and insufficient public participation in environmental protection supervision among residents in central and western China, leading to an insignificant effect of public participation on tourism carbon emissions [65]. In order to encourage the public to actively participate in environmental governance, the government should strengthen the publicity and education of environmental protection, improve the public awareness of environmental protection, and enable the public to actively participate in the supervision of enterprises’ pollution behavior [66]. Additionally, government agencies should improve the information disclosure system to protect the public’s right to know about environmental quality, so as to enhance the enthusiasm of people to participate in environmental governance.

Only two types of environmental regulations in western China (command and control, and market incentives) had significant inhibitory effects on tourism carbon emissions. Their coefficients were significantly larger than those in eastern and central China. The impacts of supervisory management and public participation on tourism carbon emissions were not significant, although the western region has made remarkable progress in building environmental infrastructure. However, tourism enterprises in western China are relatively scattered due to the large area and sparse population. In addition, there is still a substantial effectiveness gap between government environmental supervision departments and those in the central and eastern regions. Therefore, the influence coefficient of environmental regulation on tourism carbon emission was not significant. In conclusion, there were significant differences in the impacts of environmental regulations on tourism carbon emissions in the three regions of China.

## 7. Conclusions

Based on panel data from 31 provinces in China from 1999 to 2018, this research divided environmental regulations into four types: command and control, supervisory management, economic incentives, and public participation, and empirically examined the intensity, time-lag effects, and regional differences of the four types of environmental regulations on tourism carbon emissions. The findings result in three major conclusions being drawn.

Environmental regulations had significant inhibitory effects on tourism carbon emissions, but different environmental regulations had significantly dissimilar influences. The carbon emission reduction of the tourism sector in China is dominated by government regulations. Environmental regulation based on supervisory management had the most significant impact on tourism carbon emissions, followed by market incentives and command and control. Public participation had the least influence on tourism carbon emissions. A comparison of multiple balanced short panel data models showed that the baseline regression results were robust and reliable.

Environmental regulations had significant time lag effects on tourism carbon emissions. Command and control environmental regulations significantly impacted tourism carbon emissions in the first, second, third, and fourth periods in terms of lag effect strength. However, in the fifth period, they did not have a positive and significant effect. The lag of market incentives from periods one to five significantly impacted tourism carbon emissions. From the third stage, the supervisory management environmental regulation did not have significant impacts on tourism carbon emissions. The lags of environmental regulation based on public participation significantly impacted the carbon emissions of tourism in the first, second, and third phases. However, they did not play a positive and significant role in the fourth stage and beyond. Therefore, the impact on tourism carbon emission intensity was much lower than the other three types of regulations. From the decay rate of different lag periods on tourism carbon emissions, the order for the four environmental regulations was market incentives, supervisory management, command and control, and public participation. Environmental regulations strongly affected tourism carbon emissions for relatively long durations.

Environmental regulations demonstrated significantly different regional effects on tourism carbon emissions. The carbon emissions of tourism in eastern China were affected by the four environmental regulation types (command control, supervisory management, market incentives, and public participation). In contrast to the eastern region, the central region’s tourism carbon emissions were only affected by three types of environmental regulations (command control, supervisory management, and market incentives), while public participation environmental regulations had no significant impact on tourism carbon emissions. Only command control and market incentives regulations had significant effects on tourism carbon emissions in western China. Their coefficients were more significant than those in eastern and central China. The impact of supervisory management and public participation on tourism carbon emissions in western China was not substantial, but the coefficients were negative.

## 8. Implications

Based on these conclusions, the following three implications were derived. First, regional development levels must be considered to rationally formulate and select the most appropriate environmental regulation tools. All four environmental regulations in eastern China have significant inhibitory effects on tourism carbon emissions. However, only three environmental regulations in central China and only two in western China were effective. In the process of introducing environmental regulation in various regions, a blanket generalization should be avoided and close attention should be paid to optimizing the combination of environmental regulation tools. For example, tourism enterprises are widely distributed in the central and western regions where the economic foundation is not as strong. Therefore, market incentives regulation tools should be the main strategy, with command and control type regulations in an auxiliary role. Additionally, public participation regulations should be strengthened [67]. The central and western regions must continue to enhance resident awareness of environmental protection. Public education on garbage classification, energy savings, and consumption reduction measures in tourism will enhance the awareness of carbon emission targets.

Greater importance must be accorded to the time-lag effects of environmental regulations on tourism carbon emissions with more focus on the long-term impacts of policy implementation. To strengthen the restraining effects of environmental regulations on tourism carbon emissions, attention needs to be given to the current effects of environmental regulations and the time-delay effects. We should actively promote long-term and larger space dimension environmental regulations to reduce carbon emissions in the tourism industry.

Finally, the inhibitory effect of public participation in environmental regulation requires greater attention. When implementing carbon emission reduction policies, we should give full play to the role of public participation and make the public actively participate in the supervision of tourism carbon emission reduction. Governments should publicize environmental protection, improve public and enterprise awareness, give full play to the role of the public in the supervision of tourism carbon emissions, and encourage the public to actively participate in the process of environmental protection. Governments must establish and improve environmental information disclosure systems, broaden the channels for the public to disclose and report environmental problems, reduce the costs of public participation, and make environmental regulations based on public participation an important tool for lowering tourism carbon emissions. Additionally, governments should enrich the means of environmental regulation and gradually realize their transformation from command and control to public participation to maximize the incentive effect on tourism carbon emission reduction.

It should be noted that this research examined the impact of four types of environmental regulations on tourism carbon emissions from the perspective of institutional effectiveness, and drew meaningful conclusions and policy implications. However, there were some research limitations: (1) Considering the availability of data, this investigation examined 31 provinces of China and the results are at the provincial level; municipalities were not included and are still lacking in such analysis; (2) the impact of environmental regulations on tourism carbon emissions may have a spatial effect, but this research did not account for this spatial perspective. These problems are further discussed as directions for future research.

## Figures and Tables

**Table 1:** Descriptive statistics.

Variable	Variable Name	Obs	Mean	Std. Dev.	Min	Max
$TCE_{it}$	Tourism carbon emissions	620	3.37	1.49	−0.84	6.19
CC	Command control environmental regulations	620	3.04	2.05	−1.96	10.20
MI	Market incentives environmental regulations	620	22.51	1.73	17.94	25.99
SM	Supervisory management environmental regulations	620	22.93	2.82	17.89	35.54
PP	Public participation environmental regulations	620	8.92	1.61	4.93	11.12
TI	Tourism income	620	7.71	0.89	6.41	9.00
ET	Employment in tertiary industries	620	9.02	0.44	7.96	9.74
PV	Passenger volumes	620	4.02	0.80	−0.72	4.61

Note: the logarithms of the variables have been considered in the table.

**Table 2:** Baseline regression results.

Variable	$TCE_{it}$				
	(1)	(2)	(3)	(4)	(5)
CC	−0.16 ***				−0.04 ***
	(−7.49)				(−3.18)
MI		−2.64 ***			−1.60 ***
		(−12.70)			(−10.31)
SM			−0.25 ***		−0.12 ***
			(−4.56)		(−2.64)
PP				−0.07 ***	−0.03 *
				(−3.64)	(−1.95)
TI	0.51 ***	0.07	0.44 ***	0.52 ***	0.30 ***
	(5.73)	(1.03)	(9.86)	(11.92)	(4.90)
ET	0.18 ***	−0.02	0.11 ***	0.13 ***	0.05 *
	(4.13)	(−0.68)	(4.32)	(5.09)	(1.95)
PV	−0.03	0.03	0.03	0.03	−0.21 ***
	(−0.27)	(0.57)	(0.76)	(0.95)	(−2.78)
C	6.18 ***	−15.87 ***	6.53 ***	7.13 ***	−6.91 ***
	(107.20)	(−9.01)	(333.53)	(46.84)	(−5.17)
Time fixed	Y	Y	Y	Y	Y
Individual fixed	Y	Y	Y	Y	Y
N	283	313	273	283	250
$R^{2}$	0.49	0.54	0.42	0.49	0.68

Note: *t*-statistics in parentheses; * *p* < 0.1, ** *p* < 0.05, *** *p* < 0.01.

**Table 3:** Model robustness test.

Variable	$TCE_{it}$				
	OLS	$FE_{robust}$	$FE_{TW}\;$	*RE*	*BE*
CC	−0.06 ***	−0.01	−0.04 **	−0.06 ***	−0.04 ***
	(−4.05)	(−0.69)	(−2.14)	(−4.05)	(−3.18)
MI	−0.90 ***	−0.39	−1.60 ***	−0.90 ***	−1.60 ***
	(−10.13)	(−1.33)	(−5.16)	(−10.13)	(−10.31)
SM	−0.18 ***	−0.08 ***	−0.12 ***	−0.18 ***	−0.12 ***
	(−3.90)	(−2.92)	(−2.98)	(−3.90)	(−2.64)
PP	−0.04 **	−0.01	−0.03 **	−0.04 **	−0.03 *
	(−2.15)	(−0.50)	(−2.11)	(−2.15)	(−1.95)
TI	0.28 ***	0.09	0.30 ***	0.28 ***	0.30 ***
	(4.82)	(1.50)	(4.07)	(4.82)	(4.90)
ET	0.09 ***	0.05 **	0.05 *	0.09 ***	0.05 *
	(3.31)	(2.33)	(2.03)	(3.31)	(1.95)
PV	0.00	−0.15 **	−0.21 ***	0.00	−0.21 ***
	(0.00)	(−2.52)	(−2.86)	(0.00)	(−2.78)
C	−0.94	3.10	−6.91 **	−0.94	−6.91 ***
	(−1.25)	(1.25)	(−2.63)	(−1.25)	(−5.17)
N	250	250	250	250	250
$R^{2}$	0.55	0.80	0.68	0.65	0.68

Note: *t* statistics in parentheses; * *p* < 0.1, ** *p* < 0.05, *** *p* < 0.01.

**Table 4:** Impacts of environmental regulations on tourism carbon emissions with different lag periods.

Variable	$TCE_{it}$				
	Lag1	Lag2	Lag3	Lag4	Lag5
CC	−0.07 ***	−0.06 ***	−0.04 ***	−0.02 **	−0.02
	(−4.81)	(−5.67)	(−3.84)	(−2.15)	(−1.62)
MI	−1.41 ***	−1.20 ***	−1.19 ***	−1.17 ***	−0.90 ***
	(−8.73)	(−9.13)	(−8.26)	(−8.30)	(−5.65)
SM	−0.11 **	−0.09 **	−0.06	−0.01	0.00
	(−2.36)	(−2.30)	(−1.65)	(−0.16)	(0.07)
PP	−0.04 **	−0.04 ***	−0.03 *	−0.01	0.00
	(−2.24)	(−2.80)	(−1.96)	(−0.52)	(0.33)
TI	0.17 ***	0.11 ***	0.09 ***	0.10 ***	0.10 ***
	(3.93)	(3.21)	(2.62)	(2.91)	(2.83)
ET	0.04	−0.00	0.02	0.04	0.01
	(0.97)	(−0.06)	(0.49)	(1.03)	(0.34)
PV	−0.01	0.00	0.04 **	0.03 *	0.03 *
	(−0.43)	(0.20)	(2.06)	(1.85)	(1.93)
C	−5.26 ***	−3.38 ***	−3.28 ***	−3.19 ***	−0.91
	(−3.75)	(−2.98)	(−2.65)	(−2.62)	(−0.67)
Time fixed	Y	Y	Y	Y	Y
Individual fixed	Y	Y	Y	Y	Y
*N*	243	218	190	161	133
*R* ^2^	0.67	0.71	0.67	0.66	0.58

Note: *t* statistics in parentheses; * *p* < 0.1, ** *p* < 0.05, *** *p* < 0.01.

**Table 5:** Spatial effects of environmental regulation on tourism carbon emissions.

Variable	$\;TCE_{it}^{East}$	$TCE_{it}^{Middle}$	$TCE_{it}^{West}$
	(1)	(2)	(3)
CC	−0.06 ***	−0.04 ***	−0.06 *
	(−2.79)	(−2.94)	(−1.68)
MI	−1.69 ***	−1.13 ***	−1.81 ***
	(−9.33)	(−4.92)	(−5.77)
SM	−0.09 *	−0.13 ***	−0.05
	(−1.69)	(−2.90)	(−0.53)
PP	−0.03 *	−0.01	−0.03
	(−1.84)	(−0.48)	(−1.29)
TI	0.26 ***	0.26 ***	0.83 **
	(3.34)	(4.25)	(2.59)
ET	0.06 **	0.00	0.07 *
	(2.08)	(0.06)	(1.67)
PV	−0.19 **	−0.10	−0.92 **
	(−2.11)	(−1.49)	(−2.16)
C	−7.43 ***	−3.10	−8.57 ***
	(−4.83)	(−1.50)	(−3.28)
*N*	168	162	80
*R* ^2^	0.71	0.69	0.74

Note: *t* statistics in parentheses; * *p* < 0.1, ** *p* < 0.05, *** *p* < 0.01.

## Data Availability

Not applicable.
